# Hypersensitivity to Gadolinium-Based Contrast Media

**DOI:** 10.3389/falgy.2022.813927

**Published:** 2022-03-17

**Authors:** M. Teresa Gracia Bara, Alicia Gallardo-Higueras, Esther M. Moreno, Elena Laffond, Francisco J. Muñoz Bellido, Cristina Martin, Miriam Sobrino, Eva Macias, Sonia Arriba-Méndez, Rosita Castillo, Ignacio Davila

**Affiliations:** ^1^Allergy Service, University Hospital, Salamanca, Spain; ^2^Institute of Biomedical Research, University of Salamanca, Salamanca, Spain; ^3^Department of Biomedical and Diagnostic Sciences, Faculty of Medicine, University of Salamanca, Salamanca, Spain; ^4^RETIC Asma, Reacciones Adversas y Alérgicas (ARADYAL), Instituto de Salud Carlos III, Madrid, Spain

**Keywords:** gadolinium based contrast agent, allergy, immediate reactions, non-immediate reactions, hypersensitivity, skin test

## Abstract

Gadolinium-based contrast agents (GBCAs) are frequently used in magnetic resonance imaging (MRI) examinations to increase sensitivity in diagnoses. Recently, an increase in the description of hypersensitivity reactions to GBCAs has been detected. We performed research in PubMed, PubMed, SCOPUS, and EMBASE until September 2021, searching for studies regarding immediate and delayed hypersensitivity reactions to gadolinium-based contrast agents in which an allergy study was performed. The initial research identified 149 articles written in English. After excluding articles duplicated and articles that had irrelevant designs, 26 articles were included. Finally, 17 studies concerning immediate reactions, six studies concerning non-immediate reactions, and three concerning both that performed allergy evaluations were selected. In the review, we analyzed the characteristics of immediate and delayed reactions and the results of the allergy study and cross-reactivity. Skin tests seem to have acceptable accuracy, but drug provocation tests are still needed when skin tests are negative o to find alternative agents. Although cross-reactivity patterns are not well established, cross-reactivity seems to exist among macrocyclic agents. Notwithstanding, the number of patients analyzed is low and further studies are required. A management algorithm is suggested.

## Introduction

Gadolinium-based contrast agents (GBCAs) are used in radiology to increase the sensibility and specificity of magnetic resonance imaging (MRI) examinations. After approval of gadopentetate dimeglumine by the US Food and Drug Administration (FDA) in 1988, the use of GBCAs has multiplied, aiding lesion depiction and therapeutic guidance in over 500 million patients worldwide (1).

**Figure 1:**
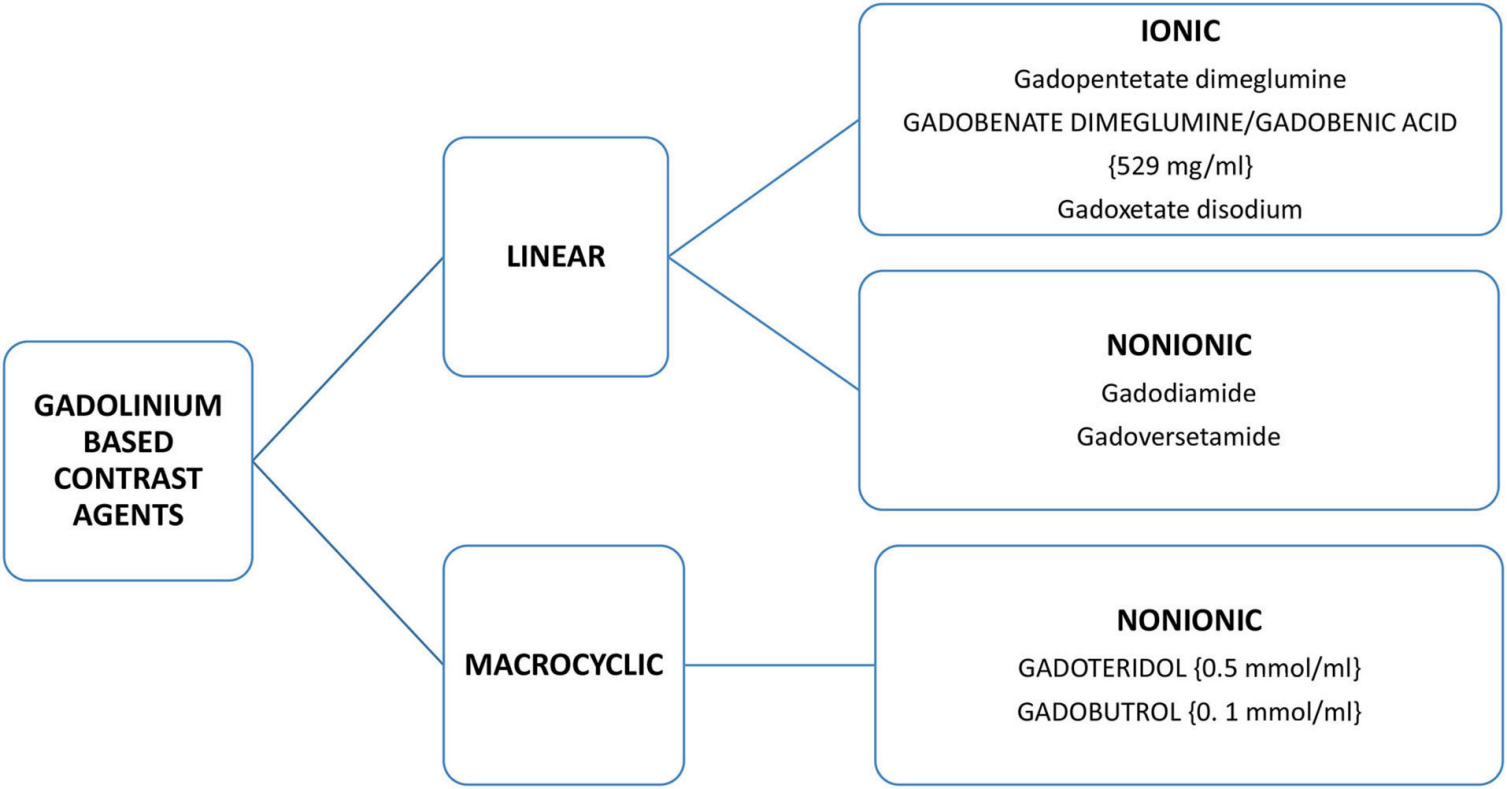
Classification of gadolinium based contrast agents (GBCAs)(-concentration of available GBCAs-).

As gadolinium administered directly is toxic, all GBCAs add to their composition a chelating substance of linear or cyclic morphology that binds to gadolinium and improves the stability, solubility, and safety of the central gadolinium heavy metal ion. Thus, GBCAs are categorized as linear or macrocyclic based on the molecular structure of the organic ligand and as non-ionic or ionic based on their net charge in solution (2) (Figure 1; Table 1). The most used GBCAs are extracellularly distributed and mainly eliminated via the kidneys, with some liver excretion demonstrated for a few agents.

**Table 1:** Available Gadolinium-Based Contrast Agents (GBCAs)and their chemical structures.

**Name and type of GBCA**	**Chemical structure**
Gadobenate dimeglumine/gadobenic acid Linear ionic	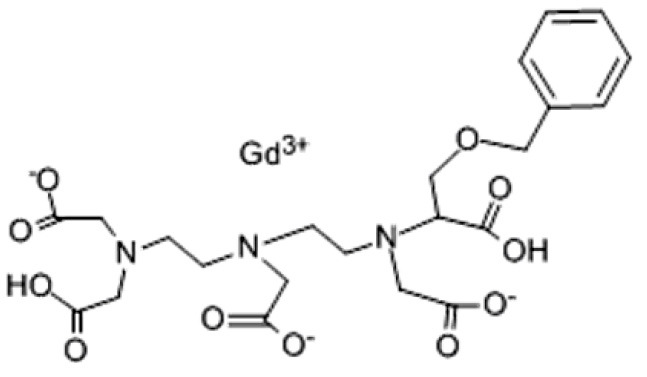
	C22H28N3O11.Gd
Gadoterate meglumine/gadoteric acid Macrocyclic ionic	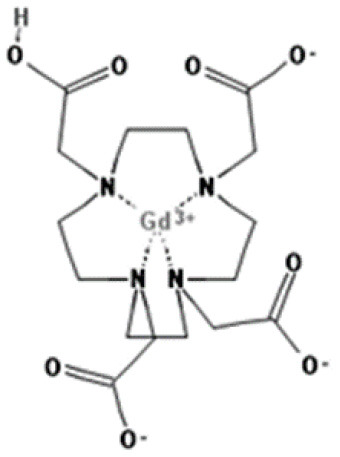
	C16 H25 Gd N4O8
Gadoteridol Macrocyclic nonionic	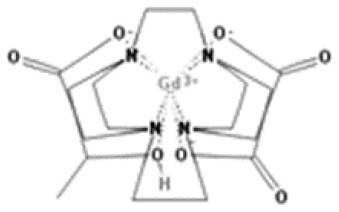
	C17 H29 Gd N4 O7
Gadobutrol acrocyclic nonionic	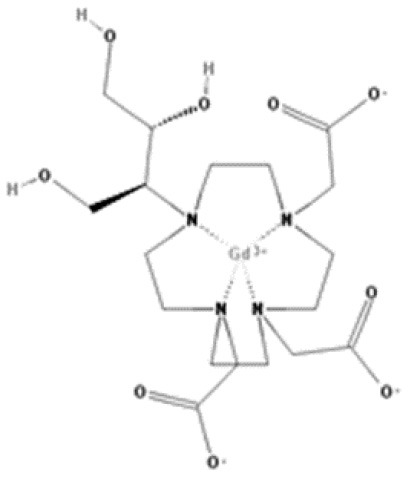
	C18 H31 Gd N4 O9

The adverse event rate for GBCAs administered at clinical doses (0.1–0.2 mmol/kg) ranges from 0.07 to 2.4% (3). Most adverse events are type A, generally mild reactions, including coldness, warmth, or pain at the injection site; nausea, vomiting; headache; paresthesias; dizziness; myalgia and arthralgia; and thrombophlebitis. If the patients are also in a situation of great stress, it is not infrequent that they can suffer a vasovagal syncope (bradycardia, hypotension, paleness, weakness, nausea, vomiting, and sweating), making it difficult to distinguish it from an allergic reaction. Type A reactions are probably caused by the direct toxicity of the contrast on the cell due to increased osmolality (concentration of solutes in a fluid evaluating the number of particles per weight -kilo- of fluid).

Hypersensitivity reactions (HRs) are uncommon and vary in frequency from 0.004 to −0.7% (4). They can be classified as immediate and non-immediate. Immediate hypersensitivity reactions (IHRs) are urticaria, angioedema, rhinitis, conjunctivitis, bronchospasm, gastrointestinal symptoms, anaphylaxis, or anaphylactic shock occurring within 1–6 h after drug administration. Non-immediate hypersensitivity reactions (NIHRs) include delayed urticaria, maculopapular eruptions, fixed drug eruptions, vasculitis, and Severe Cutaneous Adverse Reactions (SCAR); they may occur at any time as from 1 h after the initial drug administration (5).

The most frequent reactions are immediate reactions where skin manifestations are presented in 75–100% of cases, including urticaria, rashes, pruritus, and limited facial edema (6). Anaphylaxis occurs in 0.01% of cases (7). The death rate due to gadolinium contrast-induced anaphylaxis is 0.0019% and of the total deaths reported by the FDA is 0.00008%. In 2015, a case was published of a 66-year-old patient who underwent a gadoteridol scan and, upon completion of the infusion, immediately began with dry cough and facial erythema followed by seizures and dyspnea, with hypotension and ventricular fibrillation and subsequent death. Postmortem serum tryptase levels of 1,220 mcg/liter were found, suggesting a massive tryptase release during anaphylaxis (8).

In immediate hypersensitivity reactions, whether IgE mediated or not, the release by mast cells and basophils of histamine and other mediators can give rise to cutaneous manifestations, nasal congestion, dyspnea, bronchospasm, nausea, vomiting, diarrhea, hypotension, and tachycardia. In non-IgE-mediated cell activation, the mechanisms causing this degranulation include the direct effect of the contrast on the cell membrane (related to the chemical structure of the contrast and osmolality), the activation of the complement (anaphylatoxins, C3a and C5a, which are capable of activating mast cells by binding to membrane receptors other than those of IgE) and the generation of bradykinin. Considering the risk factors for developing immediate adverse reactions to GBCAs, the pattern is similar to the reactions that appear after exposure to iodinated contrast media (ICM), female gender being a predisposing factor for developing immediate hypersensitivity reactions with GBCAs (9). Although these differences are unexplained, animal studies suggest that specific sex hormones may be related to the increased incidence in females (10). Antecedents of allergy and history of a previous reaction to GBCAs are relevant determinant factors. In the analyzed studies, age and concomitant treatments were not found to be risk factors, even if other studies reported them as such (6).

Concerning non-immediate or delayed hypersensitivity reactions to GBCAs, Power et al. (11) reported an incidence of 0.05%. The reported symptoms were urticaria (66%), rash (33%), and pruritus (6.6%). Delayed reactions appeared on the same day in 46% of cases, on the following day in 20% of cases, and in 33% of cases, the moment of manifestation was uncertain (11).

Most of the publications that address an allergy evaluation did not appear until the 2000s, and most describe cases of immediate hypersensitivity. The allergic assessment of patients who have suffered a suspected hypersensitivity reaction has shown the involvement of specific immunological mechanisms, IgE- or T cells-mediated, in a significant percentage of patients. This allergy evaluation could also be helpful for the study of cross-reactivity patterns.

The study aimed to examine through a systematic review of the studies published regarding the allergic evaluation of immediate and delayed hypersensitivity reactions after the administration of GBCAs used for MRI. Another objective was to analyze the cross-reactivity patterns between GBCAs in both immediate and non-immediate reactions.

## Methods

### Literature Search

A bibliographic search on studies was performed, including the available scientific evidence up to September 2021. The primary sources for the search were PubMed, SCOPUS, and EMBASE.

We performed all possible combinations between the following keywords: Gadolinium-based contrast media/agent, gadopentetate dimeglumine, gadobenate dimeglumine, gadoxetate disodium, gadodiamide, gadoversetamide, gadoterate meglumine, gadoteric acid, gadoteridol, gadobutrol, drug hypersensitivity, immediate allergic reactions, non-immediate allergic reactions, skin hypersensitivity. After analyzing the results, only studies relevant to the subject were included.

### Inclusion and Exclusion Criteria

- Original articles, cases reports, case series, and systematic reviews were selected.
- Only articles in English were considered.
- Only articles explicitly dealing with hypersensitivity reactions were included.
- No age restriction was considered.
- At least two blinded researchers independently reviewed titles and abstracts from the initial search, and eligibility criteria determined their inclusion or exclusion.

Studies about the allergic evaluation in suspected immediate and delayed hypersensitivity reactions were prioritized. After analyzing the reviewed articles, reactions were classified as IHRs or NIHRs according to the criteria mentioned above (5).

## Results

**Figure 2:**
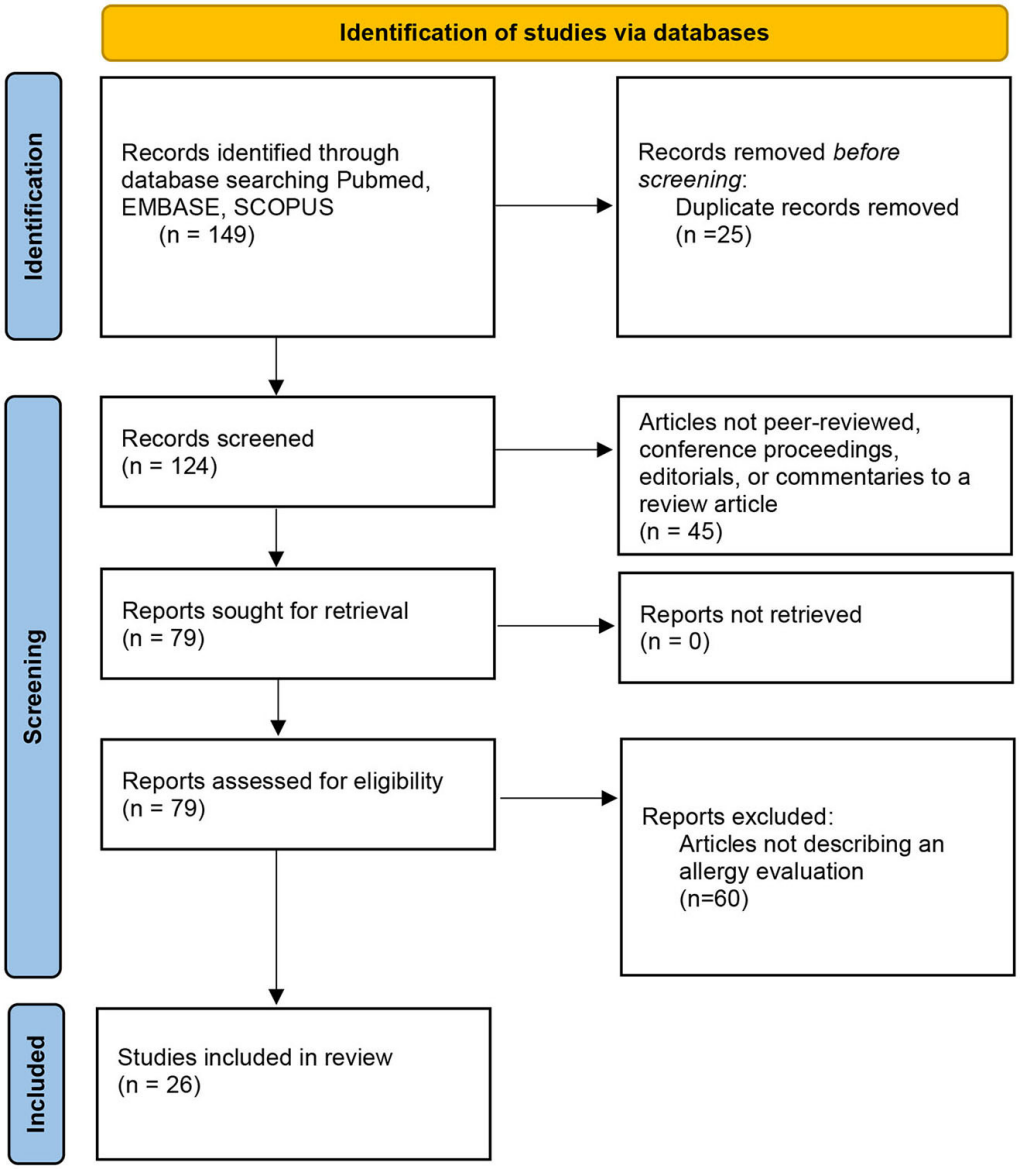
Flow diagram for search methods.

Initially, database searches showed 149 results. Articles not peer-reviewed, conference proceedings, editorials, or commentaries to review articles were excluded after abstracts were evaluated. After applying inclusion and exclusion criteria, 26 articles were included in the review (Figure 2).

In most reviewed studies, the diagnosis of hypersensitivity to GBCAs was made based on the clinical history and skin test results (ST). Skin tests were usually performed following the EAACI-ENDA methodological recommendations (12). Most manuscripts refer to previously published guidelines concerning the positivity or negativity criteria, mainly that of ENDA's guideline (12).

Nineteen articles that evaluated immediate reactions and performed an allergy evaluation were found in the bibliographic search (3, 7, 13–29). Most of them were isolated cases or small series of cases (Table 2). In all of them, skin tests were carried out with the eliciting contrast media; in some of them, evaluation with contrast media different than those that had originated the reaction was carried out. The quality of the study was considered acceptable if STs and controlled drug provocation tests (DPTs) had been performed and suboptimal if DPTs had not been performed. Whether DPTs were performed is specified in Tables 2, 3.

**Table 2:** Immediate hypersensitivity reactions to GBCAs.

**Study**	**Sample size**	**GBCAs**	**Reaction**	**Allergy study**		
				**Positive skin tests**	**Positive DPTs/Re-exposed**	**Negative DPTs/Re-exposed (well tolerated)**
Beaudouin et al. (13) Case report	1	Gadoterate meglumine (1)	Anaphylaxis (1)	Gadoterate meglumine (1)	Not performed	
Schiavino et al. (14) Case report	1	Gadopentetate dimeglumine (1)	Anaphylaxis (1)	Gadopentetate dimeglumine (1)	Not performed	
Hasdenteufel et al. (15)* Case reports	2	Gadoterate meglumine (2)	Anaphylaxis (2)	Gadoterate meglumine (2)	Not performed	
Kalogeromitros et al. (16)* Case report	1	Gadobenate dimeglumine (1)	Anaphylaxis (1)	Gadobenate dimeglumine (1)	Not performed	
Galera et al. (17)* Case reports	2	Gadobenate dimeglumine (1) Gadoteridol (1)	Anaphylaxis (2)	Gadobenate dimeglumine (1) Gadoteridol (1)	Not performed	
Moulin et al. (18) Case report	1	Gadoterate meglumine (1)	Anaphylaxis (1)	Gadoterate meglumine (1)	0	Gadobenate dimeglumine
Chiriac et al. (19)*	27	Gadobenate dimeglumine (2) Gadoteridol (4)	Dyspnea (2) Urticaria (3) Maculopapular exantema (1) Angioedema (1) Anaphylaxis (3) Facial erythema (1)	Gadobenate dimeglumine (1)	0	Gadopentetate dimeglumine(3) Gadobenate dimeglumine (1) Gadoterate meglumine (4) Gadoteridol (3)
Tomás et al. (7) Case reports	2	Gadopentetate dimeglumine (1) Gadoteridol (1)	Urticaria (1) Generalized rash (1)	Gadoxetate disodium (1) Gadobutrol (1) Gadoteridol (1)	0	Gadoteridol (1) Gadobenate dimeglumine (1)
Raisch et al. (20) Retrospective database analysis of the FAERS data/literature review	628	Gadopentetate dimeglumine (268) Gadobenate dimeglumine (178) Gadoxetate disodium (5) Gadodiamide (40) Gadoversetamide (4) Gadoterate meglumine (10) Gadobutrol (15) Gadoteridol (108)	Anaphylaxis (100%)	Gadobenate dimeglumine (2) Gadoterate meglumine (3) Gadobutrol (2) Gadoteridol (2)	Not performed	
Sellaturay et al. (21) Case reports	2	Gadobutrol (2)	Anaphylaxis (2)	Gadobutrol (2)	0	Gadopentetate dimeglumine(1)
Kolenda et al. (22)*	33	Gadobenate dimeglumine (19) Gadoterate meglumine (6) Gadobutrol (5)	Not specified	Gadobenate dimeglumine (14) Gadoterate meglumine (4) Gadobutrol (7)	0	Gadobenate dimeglumine (5) Gadoterate meglumine (10) Gadobutrol (6)
Harr et al. (23) Case report	2	Unknown	Anaphylaxis (1)	Gadoterate meglumine (1) Gadobutrol (1)	Not performed	
Clement et al. (24)* Prospective	36	Gadopentetate dimeglumine (11) Gadobenate dimeglumine (8) Gadodiamide (2) Gadoterate meglumine (10) Gadoteridol (5)	Generalized erythema (1) Extended urticaria (1) Angioedema (2) Anaphylaxis (12)	Gadopentetate dimeglumine (11) Gadobenate meglumine (8) Gadodiamide (2) Gadoterate meglumine (10) Gadoteridol (5)	Not performed	
Moreno-Escobosa et al. (25) Case report	1	Gadobutrol (1)	Anaphylaxis (1)	Gadobenate dimeglumine (1) Gadodiamide (1) Gadoterate meglumine (1)	Gadoteridol (1)	0
Seta et al. (3)* Retrospective	14	Gadobenate dimeglumine (1) Gadoterate meglumine (6) Gadobutrol (4) Gadoteridol (1)	Anaphylaxis (7) Urticaria (4) Angioedema (1) Faintness (1) Hypotension (1)	Gadobutrol (2) Gadoteridol (1) Gadoterate meglumine (1)	Gadoterate meglumine (2)	Gadoterate meglumine (11) Gadopentetate dimeglumine (1)
Hojreh et al. (26) Retrospective, cross-sectional study	17	Gadobenate dimeglumine (2) Gadoterate meglumine (13) Gadobutrol (1) Gadoteridol (2) Unknown (1)	Flushing, nausea, vomiting, urticaria (16) 84,2% Intermittent respiratory complaints (1) 5,2% Convulsions (2) 10,5%	0	0	Gadobenate dimeglumine (1) Gadoterate meglumine (7) Gadobutrol (9)
Mankouri et al. (27)* Single-center retrospective analysis	132	Gadobenate dimeglumine (18) Gadodiamide (2) Gadoterate meglumine (47) Gadobutrol (7) Gadoteridol (3) Unknown (56)	Urticaria (41) Maculopapular exantema (24) Anaphylaxis (53) Bronchospasm (6) Others/Unknown (8)	Gadobenate dimeglumine (4) Gadoterate meglumine (7) Gadobutrol (4) Gadoteridol (2)	0	Gadodiamide(1) Gadoterate meglumine (4) Gadobutrol (1)
Nucera et al. (28)*	4	Gadopentetate dimeglumine (1) Gadobenate dimeglumine (2) Gadoteridol (1)	Angioedema (1) Urticaria/Angioedema (1) Anaphylaxis (2)	Gadopentetate dimeglumine (1) Gadobenate dimeglumine (2) Gadoterate meglumine (1) Gadobutrol (1) Gadoteridol (1)	Not performed	
Gallardo et al. (29) Retrospective	5	Gadobutrol (5)	Urticaria (2) Anaphylaxis (3)	Gadoterate meglumine (2) Gadobutrol (4)	Gadobutrol (1)	Gadoxetate disodium (1) Gadoterate meglumine (2)

* *Positivity criteria according to ENDA's guidelines*.

*The rest of the studies do not specify the positivity criteria, except (26) that follow the European Society of Contact Dermatitis recommendations*.

**Table 3:** Non-immediatehypersensitivity reactions to GBCAs.

**Study**	**Sample size**	**GBCAs**	**Reaction**	**ST**	**DPT**
Power et al. (11) Observational	30373 doses	Gadobutrol	15 delayed reactions, 0.049 % (15.6% of 96 total reactions)	Not performed	Not performed
Nagai et al. (30) Case report	1	Gadobutrol	Rash maculopapular	PT + Gadobutrol	Not performed
Bordel et al. (31) Case report	1	Gadobutrol	Pustulosis	PT + Gadobutrol	Not performed
Seta et al. (3)* Retrospective	14 (1 delayed)	Gadoteric acid	Exanthema+dyspnea	PT and IDTs -	Positive 1 ml of gadoteric acid
Boehm et al. (32) Case report	1	Gadobutrol	Exanthema+cardiac symptoms	Not performed	Not performed
Mankouri et al. (27)*	132 p	Gadoteric acid 35.3% Gadobenic acid 13.5% unknow 42.1%	Delayed reaction (22) (16.7%)	1 ST +Gadoteridol	Not performed
Gallardo et al. (36) Case report	1	Gadobutrol	Exanthema maculopapular	PT and IDTs -	Positive Gadobutrol Negative Gadoxetate disodium
Gauthier et al. (33)* Case report	1	unknown and Gadobutrol	Febrile macular exanthema and purpuric lesions	IDT + Gadoteridol	Not performed
Macias et al. (34) Case report	1	Gadobutrol	DRESS	IDT + Gadoxetate disodium	Positive Gadoteric acid

*IDT, intradermal test; ST, skin test; DPT, drug provocation test; Ref, references; P, patients*.

* *Positivity criteria according to ENDA's guidelines*.

*The rest of the studies do not specify the positivity criteria, except (30) that follows the Contact Dermatitis Research Group Scoring system recommendations and (31) that follow the European Society of Contact Dermatitis recommendations*.

In IHRs, in most articles, skin prick tests (SPTs) with undiluted commercial solutions and intradermal tests (IDTs) diluted at 1:10 were generally used (3, 17, 19, 25, 29), actual concentrations were not mentioned in the articles. In the case of severe reactions, IDTs began with higher dilutions (35). No study specifies the concentration used for skin testing; they only set the dilutions. That is an omission that has to be remarked in this review. STs were performed with the GBCAs involved in the reaction if known, although some authors recommend using a panel of GBCAs (28, 29).

In addition, we found nine studies that included NIHRs (3, 11, 27, 30–34, 36) (Table 3). In NIHRs, intradermal tests were performed at a 1:10 dilution and patch tests (PTs) with undiluted GBCAs. When an IDT test result was negative at the 1:10 dilution, some authors have performed IDT tests with the undiluted GBCAs (33).

In some studies, when STs were negative, controlled provocation tests were performed (3, 7, 18, 19, 21, 22, 25–27, 29, 34, 36). There are very few articles specifying the doses and schedules used for DPTs. Generally speaking, we found no consensus regarding total doses or intervals. Doses for DPTS ranged from 1 to 10 mL (3, 29, 36), with increasing doses at 1-h intervals (29, 36). In some of them, patients tolerated another GBCA than those they were sensitized to (3, 7, 18, 21, 26, 27, 29, 36).

## Discussion

### Immediate Reactions

GBCAs' immediate reactions have been infrequently described, with isolated case reports or short case series published. The first case of severe anaphylaxis following the administration of gadolinium contrast, gadopentetate dimeglumine, was described in 1990 by Weiss (37). Thereafter, an increasing number of IHRs have been described. The most frequent symptoms were erythema, maculopapular exanthema, urticaria, angioedema bronchospasm, or other respiratory symptoms. In severe cases, anaphylactic shock can occur.

Studies suggesting specific IgE-mediated immunological mechanisms in immediate reactions with positive skin test results with GBCAs have been reported (3, 7, 13–29) (Table 2). The first case with positive SPTs and IDTs was published in 2003, after anaphylaxis to gadopentetate dimeglumine (14). In 2018, a French group published the first prospective multicenter study to explore IHRs to iodinated and gadolinium-based contrast agents (24). They evaluated 36 patients with prior hypersensitivity reactions to GBCAs. Ten patients in the trial had true IgE–mediated allergy to the culprit GBCAs, and these patients often had more severe index IHRs (24).

Hasdenteufel et al. (15) reported two anaphylactic shocks with gadoterate meglumine. Both patients had positive SPTs and IDTs with gadoterate meglumine but negative with three linear GBCAs. Galera et al. (17) described two anaphylaxis, one with gadoteridol and another with gadobenate dimeglumine. In both cases, STs were positive only with the culprit GBCAs. In a recent study (29), our group presented five patients with immediate reactions to gadobutrol; 4 out of 5 had positive STs with the culprit GBCA, and two positive STs with another macrocyclic agent. When evaluating immediate reactions to GBCAs, SPTs were safer but had lower sensitivity than IDTs. Nonetheless, some reported cases had positive SPTs with GBCAs (14, 15, 17, 18, 22–24). Notwithstanding, most cases diagnosed by positive STs were due to positive IDTs. Nevertheless, as skin tests' sensitivity is suboptimal, DPTs are necessary to diagnose and demonstrate tolerance to other GBCAs. The DPT is considered the gold standard for the diagnosis of drug hypersensitivity reactions (5).

In many patients, particularly in the case of severe reactions, an alternative GBCA can be tried to verify tolerance or assess reactivity. Such tests can confirm or exclude the diagnosis when there is no other available evidence and can be used to look for an alternative GBCA. Thus, Tomás et al. (7) described two cases of hypersensitivity to gadopentetate dimeglumine and gadoteridol, with negative STs and DPTs with alternative GBCAs. The first patient had a negative DPT with gadoteridol. The second case, due to gadoteridol, tolerated gadobenate dimeglumine (7). Another study (19) showed an excellent negative predictive value for GBCA skin tests in 11 patients with IHRs for 10 of them and NIHRs for one. The 11 patients re-exposed to GBCAs tolerated without reaction the GBCA that yielded a negative ST (3 of them with pretreatment). Notwithstanding, Moreno-Escobosa et al. (25) published one anaphylaxis with gadobutrol with positive STs to all studied agents (gadobenate dimeglumine, gadodiamide, and gadolinium), except gadoteridol (a macrocyclic agent). The DPT with gadoteridol triggered an immediate reaction, suggesting the need for DPTs despite negative STs.

A study showed the results of provocations with low-doses of GBCAs (3). The authors performed single-blind placebo DPTs with 1 mL of the GBCA solution (corresponding to one-tenth of the usually injected GBCA for MRI) during an 8-h hospital stay. They included 14 patients that underwent controlled exposure and skin tests with gadolinium contrasts. Twelve patients had presented immediate hypersensitivity reactions, one a delayed reaction, and another a reaction that could not be classified. A positive allergy workup was found in 5/14 patients, with positive GBCA IDTs in three cases, and positive DPTs in two cases (one immediate and one delayed). After the allergic study, 10 out of 14 patients were contacted, and two underwent a new injected GBCA using the contrast tested negatively during DPTs, without premedication. Analyzing the results, the authors calculate a negative predictive value (NPV) of skin tests of 86%. Other studies have also found a good NPV of skin tests (27, 29).

Hojreh et al. (26) did a retrospective study on 17 children who had presented a reaction with GBCAs. Twenty-one DPTs were performed in ten patients for at least two substances—either gadoterate meglumine, gadobutrol, or gadobenate dimeglumine. None of the patients who underwent DPT showed any sign of a hypersensitivity/allergic-like reaction. Two patients exhibited dizziness, nausea and flush, and one patient experienced vomiting, all categorized as chemotoxic responses but not as allergic reactions.

### Cross-Reactivity

Cross-reactivity between gadolinium chelates is still unclear. Kolenda et al. (22) described 33 patients with immediate reactions to GBCAs, finding cross-reactivities, more frequently between gadoterate meglumine and gadobutrol, both macrocyclic agents, although they described three patients monosensitized to gadobutrol. Moulin et al. (18) reported an anaphylactic reaction to gadoterate meglumine and a strongly positive SPT with gadoterate but negative STs to four GBCAs, linear and macrocyclic. They performed a DPT with gadobenate dimeglumine with good tolerance. We have described similar sensitization patterns: patients with selective responses to gadobutrol and patients sensitized to macrocyclic agents (29). Notwithstanding, the cross-reactivity between macrocyclic and linear GBCAs has not been elucidated to date, and cross-reactivity between linear agents has not been addressed. Recently, Mankouri et al. (27) found cross-reactivity in 7 of 18 allergic patients (38%). Overall, amongst the 18 patients in whom both linear and macrocyclic GBCAs were tested (either as culprit agents or alternatives), the cross-reactivity rate was 27.7% among macrocyclic agents, 5.5% among linear agents, and 5.5% between both.

A lower rate of immediate allergic adverse events has recently been reported using non-ionic, non-protein-bound agents and linear GBCAs (gadodiamide) (27, 38). However, the potential GBCA deposition in the brain observed preferentially with linear GBCAs has limited their use (39). In some countries, the linear GBCA agents gadodiamide and gadopentetate dimeglumine have been withdrawn from authorization of use by medical authorities, with difficulty to find substitutive GBCAs in case of HRs to macrocyclic GBCA.

### Premedication

It does not appear that premedication with corticosteroids prevents a future reaction. A 2019 systematic review and meta-analysis sought patients with immediate reactions to GBCAs who were undergoing repeated administration of GBCAs (40). The study concluded that in patients with a history of immediate reaction to GBCA, the repeated HRs to the same GBCA may occur in ~39%, despite the use of adequate premedication with corticosteroids. They found no difference in the rate of reactions when comparing macrocyclic with linear-ionic GBCAs (40). Other studies have also found no utility of premedication to prevent future reactions with GBCAs (26, 41–43).

### Non-immediate Reactions

Delayed reactions to GBCAs are extremely infrequent, with few cases described to date, most of them due to gadobutrol (11, 30–32, 36).

One case (31) involved an acute generalized exanthematous pustulosis (AGEP) due to gadobutrol, with PTs yielding positive results to gadobutrol on days 2 and 4 and negative results to gadoterate meglumine. DPTs were not performed. Another case involved an erythematous maculopapular rash following the administration of gadobutrol (30). PTs were performed with gadobutrol, gadoteridol, and gadoterate meglumine (all macrocyclic agents), as well as gadodiamide and gadopentetate meglumine (both linear agents), with positive results only to gadobutrol. As in the previous case, DPTs were not performed. The third case was a severe delayed reaction with cutaneous and cardiac symptoms. Neither PTs nor DPTs were performed (32).

In 2021, Gauthier et al. (33) reported a delayed reaction to GBCA, showing a positive delayed IDT with undiluted gadoteridol. DPTs were not performed. The same year our group published two case reports. A case of delayed exanthema with Gadobutrol (macrocyclic) with tolerance to gadoxetate disodium (linear) (36). A woman consulted our Allergology Service because 24 h after administration of gadobutrol developed mild itching and a generalized erythematous and generalized erythematous rash. PT and IDTs tests were negative. Given the likely need for future MRI scans, that the skin reaction had not been severe, and the diagnosis was uncertain, the patient gave written informed consent for a DPT with the usually injected gadobutrol dose for MRI, suffering a delayed rash. Subsequently, a new exposure test with a full dose of gadoxetate disodium was tolerated (36). That is the first reported case of a NIHR with gadobutrol in which a complete allergy study was performed, including DPTs, to confirm the diagnosis and offer a safe alternative for subsequent administration of GBCAs.

The second case report was a 13-year-old boy of a drug rash with eosinophilia and systemic symptoms (DRESS) syndrome in a child sensitized to chemically and antigenically unrelated substances: antibiotics, NSAIDs, and gadolinium-based contrast media (34). The boy had a reaction with gadobutrol -a macrocyclic non-ionic GBCA- and a doubtfully positive result with disodium gadoxetate, a linear ionic GBCA. Although guidelines for DRESS usually recommends not performing DPTs with the suspicious drug and structurally related drugs due to the risk of eliciting a new reaction (44), providing an alternative GBCA was considered essential for disease management by the attending physicians, so a DPT was performed with gadoteric acid. The patient developed symptoms again, which confirmed DRESS.

The main limitation of this review is that the quality of the evidence was not formally assessed, as the studies included individual patients or few patients; hence, the quality of the evidence is very low. Consequently, rates of hypersensitivity reactions to individual GBCAs are not available. Table 4 enumerates the unmet needs that should be the object of future research.

**Table 4:** Unmet needs and future research.

**Unmet needs and future research**
To improve knowledge of mechanisms involved in IHRs and NIHRs
To identify the epitopes involved in immune reactions to GBCAs
To determine sensitivity and specificity of skin tests in IHRs and NIHRs
Development of *in vitro* studies for diagnosis
To further clarify cross-reactivity among the different GBCAs
To assess the usefulness of premedication in the prevention of reactions

*IHRs, Immediate hypersensitivity reactions; NIHs, Non-immediate hypersensitivity reactions; GBCAs, Gadolinium-based contrast agents*.

## Conclusion

Hypersensitivity reactions to GBCAS are rising due to the worldwide increase in nuclear magnetic resonance diagnostic techniques using GBCAs. Although many immediate reactions after the administration of GBCAs are not genuine allergic reactions, reactions due to specific immunological mechanisms, both immediate and delayed, are increasingly being described and confirmed by positive results in skin or challenge tests. The usefulness of skin tests with GBCAs is not yet well established because most studies have included low figures of patients. Although some studies of immediate reactions after GCBA administration have suggested a high negative predictive value of STs, DPTs are still necessary to confirm or exclude the diagnosis or find alternative GBCAs.

**Figure 3:**
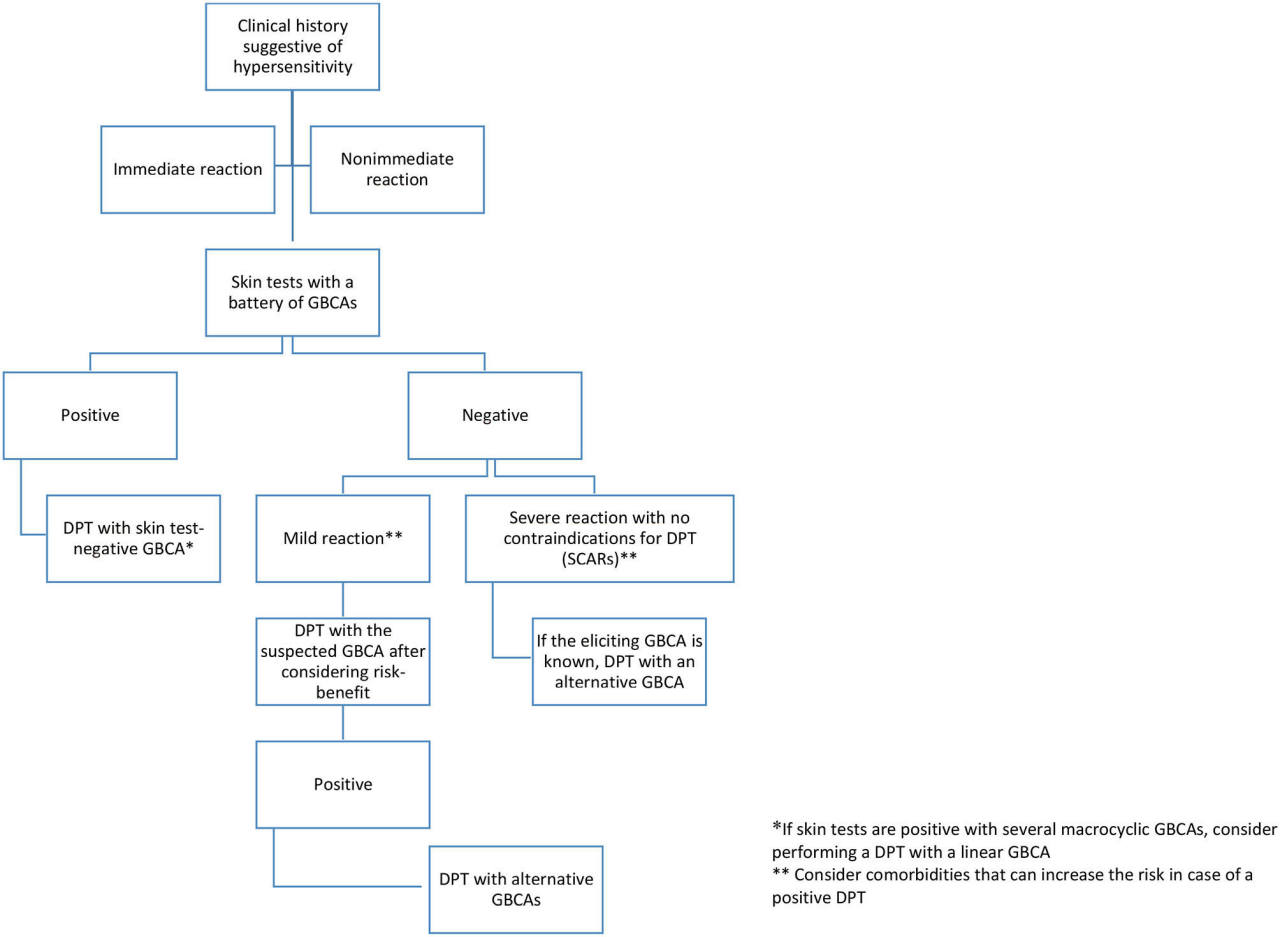
Algorithm for the diagnosis of hypersensitivity reactions to GBCAs. DPT, drug provocation test; GBCA, gadolinium-based contrast agent; SCAR, severe cutaneous reaction.

Cross-reactivity patterns between different GBCAs are not well established. Notwithstanding, data from recent studies point to a partial cross-reactivity among macrocyclic agents and no or very low cross-reactivity between macrocyclic and linear agents. Nevertheless, further studies are required to confirm these data. In Figure 3, we suggest a possible algorithm for the study and selection of GBCAs in case of purported reactions to these agents.

## Data Availability Statement

The original contributions presented in the study are included in the article/supplementary material, further inquiries can be directed to the corresponding author/s.

## Author Contributions

EMa and ID contributed to conception and design of the study. MG and AG-H organized the database and wrote the first draft of the manuscript. All authors contributed to manuscript revision, read, and approved the submitted version.

## Conflict of Interest

The authors declare that the research was conducted in the absence of any commercial or financial relationships that could be construed as a potential conflict of interest.

## Publisher's Note

All claims expressed in this article are solely those of the authors and do not necessarily represent those of their affiliated organizations, or those of the publisher, the editors and the reviewers. Any product that may be evaluated in this article, or claim that may be made by its manufacturer, is not guaranteed or endorsed by the publisher.
